# Southeast Asia in focus: stemming the reawakening of prohibitionism

**DOI:** 10.7448/IAS.19.1.21279

**Published:** 2016-06-26

**Authors:** Fifa Rahman, Adeeba Kamarulzaman

**Affiliations:** 1 Policy Department, Malaysian AIDS Council, Kuala Lumpur, Malaysia; 2 Faculty of Medicine, Centre of Excellence for Research in AIDS (CERiA), University of Malaya, Kuala Lumpur, Malaysia

In the past decade, harm reduction has slowly but steadily spread throughout the Association of Southeast Asian Nations (ASEAN) region, largely in response to the growing HIV epidemic among people who use drugs (PWUD) [1,2]. More recently, there has been a nascent realization among Southeast Asian governments of the lack of health, criminogenic and socioeconomic benefits of compulsory drug detention centres (CDDCs) [3]. These are a distinctive form of response in the name of treatment for PWUD commonly found in Cambodia, China, Indonesia, Lao PDR, Malaysia, Myanmar, Philippines, Thailand and Vietnam [3]. CDDCs are often run by public security personnel, do not have opioid substitution therapies and have up to 90% relapse rates [4]. Measures that are undertaken to treat people who use drugs within these centers run counter to accepted norms and evidence-based practices and often times violate human rights principles [4]. A recent systematic review of the scientific evidence on the effectiveness of compulsory drug treatment showed that these centres do not lead to improved outcomes with some studies suggesting potential harms associated with it [5].

Pursuant to this realization, elements of voluntary, community-based drug treatment centres began to emerge and continue to be piloted and assessed in a number of countries across Asia [3]. In addition, the visibility and/or availability of Asia-specific evidence is increasing, including studies on the return of investment and cost-efficacy of harm reduction [6] and critical analyses on the failure of compulsory drug treatment centres to address the harmful effects of drugs [7,8].

Against these positive developments, of late, a number of more punitive and prohibitionist policy changes are emerging throughout Southeast Asia, threatening to destabilize communities gaining from voluntary efforts. In Malaysia, for example, the National Anti-Drugs Agency (NADA), which implemented the lauded voluntary Cure & Care clinics, has reverted to punishment and detention of PWUD. Most recently, on 16 May 2016, the Malaysian parliament debated a legal amendment to the Dangerous Drugs Act 1952 that would enable NADA to have the same pre-trial detention powers as police [9].

NADA recently provided an illustration of its services for women who use drugs, that is, compulsory detention, and within the CDDCs, religious, cooking and hairdressing classes intended to address their drug dependence [10]. This is counterintuitive to established research on women who use drugs, including their increased risk of HIV infection due to structural, biological and behavioural reasons; traumatic child separation; multiple comorbidities; and transgenerational familial instability [11,12]. These factors suggest the need for a more nuanced, gender-sensitive and multifactorial approach to addressing their drug dependence. In 2014, 20% of new HIV infections in Malaysia were reported in women [13].

In the Philippines, the national presidential elections on 9 May 2016 resulted in a troubled political leadership, which could have implications on the current stance of Philippines being a death penalty abolitionist nation. The president-elect, Rodrigo Duterte, is a known proponent of death squads and extrajudicial killing of “criminals.” Of late, reports of killings of drug offenders are being accompanied by police assertions of the legality of their actions [14].

Meanwhile, at the time of writing, Indonesia is preparing for the next round of executions of drug offenders, despite being booed for its defence of the death penalty for drugs at the UN General Assembly Special Session on Drugs in New York in April 2016 [15]. At the same time, Indonesia continues to submit appeals for its citizens on death row for drug offences abroad, actions that seem incongruent with domestic drug policy [16]. This apparent policy incongruence has also been seen to a lesser extent in the Philippines, with Duterte stating *Wala akong magawa* (I cannot do anything) about persons sentenced to death for drug offences overseas. However, in the same interview, with regard to Mary Jane Veloso, a Filipina on death row in Indonesia, he stated that he would attempt to ask for a reprieve [17].

To prophesize catastrophic regression may be premature, but these are certainly concerning developments that academicians, the civil society, PWUD, government drugs bodies, HIV organizations and programmers and families of PWUD must take note of. These should motivate them to adopt policies that not only make economic sense but also reduce the harmful effects of drugs, improve the functionality and stability of PWUD and, above all, respect basic human rights.

From these examples, it can be gleaned that, overall, there is a burgeoning growth of punitive sentiment in the region, creating a hostile environment for further scale up of HIV prevention, treatment and diagnostic services. Progress gained in terms of an enabling environment for key affected populations with HIV via harm reduction modalities are giving way to demagoguery and injustice, and at the most difficult time, when HIV funding is dissipating. Effects of this sentiment have already seen the cancellation of operational research in Cebu, Philippines, which had a needle-and-syringe exchange programme component, due to political perception that harm reduction is “pro-illegal drugs” by placing too much importance on the reduction of HIV transmission, thereby sabotaging drug control efforts [18].

In consideration of such circumstances, would it be overly fatalistic to predict the inability to achieve the 90-90-90 goals to end AIDS? Without dogged determination to end criminalization, to scale up harm reduction and to introduce drug policy success metrics that reflect the science and human experience of key affected populations, this outcome is imminent.

The Johns Hopkins-*Lancet* Commission on Drug Policy and Health recently critically analyzed international drug policy and its impact on public health, making crucial and important recommendations. These included the need for countries to decriminalize minor non-violent drug offences like use and possession, and the need for countries to include health, development and human rights indicators in metrics to judge success of drug policies [19]. Evidence-based metrics was broken down into minute detail in a 2015 report by the Igarap Institute, which recommends that states adopt these output indicators, among others: the number of companies/firms that promote employment opportunities for former drug offenders/users; the number of media articles that attribute positions to credible scientific evidence; the number of deaths related to long-term health problems directly associated with drug use; the number of persons entering prevention/harm reduction programmes; and the number of reported cases of torture and cruel, inhuman or degrading treatment of PWUD [20].

Throughout Southeast Asia, drug policy success continues to be measured based on increased arrests of PWUD, which, if anything, only proves that there are more drug harms, more instability, more barriers to accessing HIV services, increased HIV transmission rates and more problematic drug use, as individuals remain in situations where they are in contact with law enforcement and are unable to seek voluntary treatment and welfare services.

Southeast Asian governments attending the ASEAN meetings continue as a whole to promote the futile ASEAN drug-free target, despite all evidence that eradication of all drugs is impossible [21]. The ASEAN meetings have been a unifying platform for the continuation of ineffective and expensive drug policies that have been proven to drive HIV infections. A roadmap towards constructive engagement with Southeast Asian governments is necessary to reverse the prohibitionist tide. In the spirit of the International Day against Drug Abuse and Illicit Trafficking on 26 June, there is an urgent need for the United Nations agencies, in particular the United Nations Office on Drugs and Crime (UNODC) and WHO, to think about engaging Southeast Asian nations and ASEAN as a body with these concise, evidence-based messages in a persistent and multi-pronged manner.
